# Effects of Modified-Otago Exercise Program on Four Components of Actual Balance and Perceived Balance in Healthy Older Adults

**DOI:** 10.3390/geriatrics7050088

**Published:** 2022-08-30

**Authors:** Nichapa Khumpaneid, Theerapat Phoka, Surasa Khongprasert

**Affiliations:** 1 Research Unit—Exercise Physiology in Special Population, Faculty of Sports Science, Chulalongkorn University, Bangkok 10300, Thailand; 2 Department of Chemistry, Faculty of Science, Chulalongkorn University, Bangkok 10300, Thailand

**Keywords:** falls, older adults, balance performance, OTAGO

## Abstract

Falls are a serious problem for older adults, leading to adverse injuries and decreased quality of life. Balance impairment is a key factor in falls. The Otago Exercise Program (OEP) is a promising intervention for preventing falls, thereby improving balance and gait. Previous studies reported improved effects on balance using the OEP conducted in a group setting, and recommended additional walking. Walking is a feasible exercise that benefits both fall-related physical and physiological functions. This study aims to investigate the effects of a modified-Otago Exercise Program (modified-OEP) on four components of actual balance (static, dynamic, proactive, and reactive balance) and perceived balance in healthy older adults, by conducting the modified-OEP in a groupsetting, and including additional walking in one session to gain better efficacy. Participants aged 60–85 years old were randomly assigned to the modified-OEP group or the control group (CT). The modified-OEP consisted of 60 min sessions made up of 30 min of OEP and 30 min of walking, three times a week for 12 weeks, while no intervention was assigned to the control group. The modified-OEP group showed significant improvement in the four components of actual balance and in perceived balance. Furthermore, the modified-OEP group outperformed the control group in all parameters except for dynamic balance, after 12 weeks. The present study highlights the beneficial effects of a modified-OEP on all balance components. Additionally, this study is the first to demonstrate the measurement of all actual balance components as well as perceived balance.

## 1. Introduction

Falls are one of the most prevalent health issues among older adults, with an approximate incidence rate of 30% annually [1]. Falls can cause fatal injuries, such as fractures, which increases the risk of morbidity and decreases quality of life [2]. Moreover, people who have experienced a fall are more likely to fall repeatedly [3].

Balance is a synergistic interplay between numerous physiologic and cognitive components that allows quick and precise response to a disturbance [4]. According to Shumway-Cook and Woollacott, actual balance consists of four components including static balance, dynamic balance, proactive balance, and reactive balance [5]. Balance issues are one of the most prominent disorders among older adults [6], resulting in an increased fear of falling, which is reported as a key contributor to falls [7]. Thus, improvement of balance is a promising fall prevention strategy.

Several exercise programs have been reported as effective interventions for falls prevention including balance training [1], resistant exercise [2], endurance training [3], flexibility exercises such as yoga [4], and sensorimotor exercises such as Tai Chi [5]. Despite the demonstrated benefits, the performance of these interventions differs in significant ways.

Therefore, a common exercise program targeting multi-component training would be more attractive.

The Otago Exercise Program (OEP) is one of the most popular exercise programs for preventing falls in older adults. Generally, it is designed as a home-based exercise program consisting of muscle strengthening exercises, balance training, and walking [8]. The program consists of approximately 30 min of moderate-intensity exercise conducted at least three times per week, and walking on alternate days at least twice per week is encouraged [8]. Several studies have demonstrated a positive effect of the OEP on balance and falls [9,10,11,12]. A recent meta-analysis on the effects of the OEP on actual (static, dynamic, proactive, and reactive) and perceived (fear of falls) balance reported that the OEP promoted static, dynamic, and proactive balance and reduced fear of falls [13]. The research also demonstrated that group training improved balance more effectively than individual training [13]. Although the effects of the OEP on balance have been documented, most studies focus on one or two balance components [13]. Additionally, a strong correlation between actual and perceived balance has been documented by the authors of [14]. To the best of our knowledge, no study has been done to determine the effect of the OEP on the four components of actual balance and perceived balance in one study.

Walking is a fundamental activity that anyone can accomplish because it doesn’t require any specialist skills or equipment [15]. As reviewed by Tudor-Locke et al., older adults should walk between 7000 and 10,000 steps per day to acquire the same health benefits as 30 min of moderate-to-vigorous physical activity [16]. Another suggestion is that moderate-intensity walking should be performed by older adults for 3000 steps/day or 30 min/day [17]. Walking benefits both balance components and fall-related psychological conditions [18]. Walking at a self-selected pace for 30 min, 2–3 times per week reduced fall incidence in older adults over a 16-month follow-up period, as reported by the authors of [19].

As aforementioned, walking is suggested as an optional exercise in the OEP manual. We hypothesize that incorporating walking into a single OEP session may produce greater effects than the OEP alone. To improve the efficacy of the OEP in balance components while retaining the original program’s simplicity, we have introduced a modified-OEP, which includes walking exercise and is performed in a group. Our primary objective was to determine whether such a program could enhance balance. To provide better insight into balance components, we evaluated the four components of actual balance, and perceived balance.

## 2. Materials and Methods

### 2.1. Participants

This study was a double-blinded randomized control trial conducted with participants aged between 60 and 85 years old. The inclusion criteria included: (a) Eligible for movement without using any walking aid equipment; (b) Exercise less than 150 mins per week; (c) Timed Up and Go score is more than 10 s; and (d) Urinary and bowel continence. Participants were excluded if they had neurological conditions (e.g., Parkinson’s disease, stroke), vision problems, or contagious diseases. All participants were recruited from Bangkok and nearby provinces.

**Figure 1:**
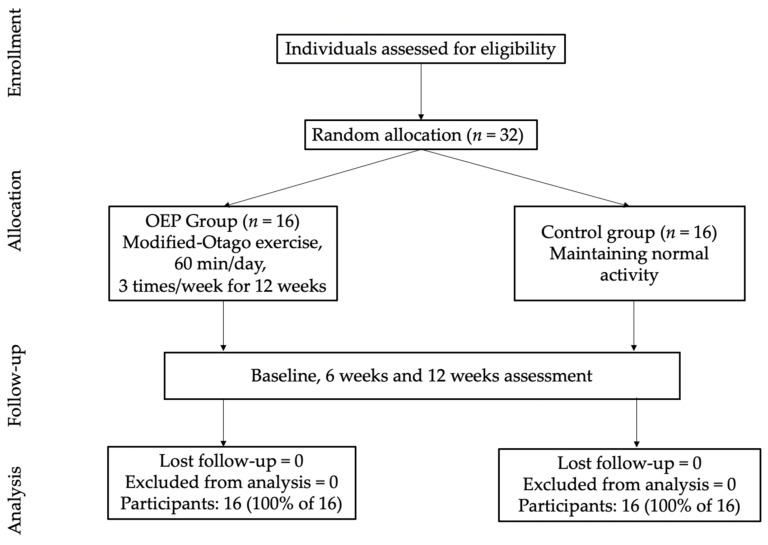
CONSORT flow diagram displaying cluster randomized clinical trial participant pathways through the 12 weeks study. OEP = Otago Exercise Program.

The sample size was calculated by G*Power version 3.1.9.4 [20]. The parameters were set as follows: effect size *t* = 0.549; alpha err prob = 0.05; power (1-B err prob) = 0.8; number of groups = 3; number of measurements = 3; and Corr among rep measures = 0.5. The total sample size after accounting for a 30% drop out rate was calculated to be 32, or 16 participants per group. A total of 32 participants were enrolled in this study. Each was randomly allocated into either the modified-Otago Exercise Program (OEP) group or the control group (CT); each group contained 16 participants. Details of the subject recruitment and allocation process are shown in Figure 1. The study protocol was approved by Chulalongkorn University Institutional Review Board, Thailand (183.1/64). All participants signed the informed consent form before starting the program. Participant characteristics are described in Table 1.

### 2.2. Intervention

To improve the efficacy of the balance improvement intervention, we modified the OEP by incorporating the additional walking, recommended by the OEP guidelines, into a single exercise session [8], and conducting it as a group exercise program in accordance with a previous meta-analysis [13]. As shown in Table 2, the modified-OEP group intervention consisted of 15 min of walking followed by a 30 min Otago Exercise Program consisting of balance and strength training, followed by another 15 min of walking. The 15 min walking was accomplished through continuous walking at an individual pace in the ~15 m training room. Participants were asked to walk 15 m, make a U-turn towards the left side, walk 15 m, then make a U-turn towards the right side at the next turning point, in order to achieve an equivalent effect on the promoted benefit of symmetry when turning left and right. This was repeated until the session was complete. Throughout the walking session, participants were encouraged by the physical therapist to continue walking at their own pace. Moreover, participants were instructed on the correct walking manner. The intervention was administered three times a week for twelve weeks, with eight individuals per session.

The control group was instructed to maintain their current level of physical activity without being assigned specific exercises.

### 2.3. Outcome Measurements

Static balance is the ability to maintain a position in the absence of disturbances, generally when seated or standing [21]. In this study, the modified Clinical Test of Sensory Interaction and Balance (mCTSIB) and the Single-leg Stance test (SLS) were used to assess static balance [22]. The mCTSIB and SLS were performed using the Biodex Balance System. (Biodex Medical System, Shirley, NY, USA, ICC = 0.76). The participants were instructed to stand still in four different sensory conditions: (a) Eyes Open—Firm Surface (EO—Firm), (b) Eyes Closed—Firm Surface (EC—Firm), (c) Eyes Open—Foam Surface (EO—Foam), and (d) Eyes Closed—Foam Surface (EC—Foam). The sway index was calculated by the machine. A Single-leg Stance test was done using the Biodex Balance System software. Participants were asked to stand for as long as possible on their preferred leg and the overall stability index (OSI) was calculated by the device. A high OSI score indicates poor balance, while a low score indicates good balance.

Dynamic balance refers to the maintenance of balance in response to internal or external perturbations [5]. The Berg Balance Scale (BBS) [23] evaluates 14 tasks including 9 dynamic and 5 static balances used for assessment.

Proactive Balance refers to the anticipation of an unexpected postural disturbance [5]. The Timed Up and Go test (TUG) was performed for assessment purposes according to the guidelines described in a previous study [24]. The participant was requested to stand up from an armless chair (46 cm height), walk 3 m at an individual speed, turn around, walk back, and sit on the chair. The time was recorded from the time the participant was asked to stand.

Reactive balance is regarded as compensating for an unanticipated postural disturbance [5]. The Limits of Stability test (LOS) was performed using the Biodex Balance System, according to the guidelines described in a previous study [25]. Briefly, participants had to lean towards a target while standing on a completely unstable platform, in order to move a cursor that was displayed on a liquid crystal display, using current model software. Participants were asked to perform the test as quickly and precisely as they could while keeping their bodies in a straight line. The LOS overall score, which consists of eight directions (forward, backward, left, right, forward-left and -right, and backward-left and -right), was calculated by the software.

Perceived balance generally measures balance confidence and fear of falling using a questionnaire, and this study employed the Falls Efficacy Scale (FES) for the assessment of perceived balance [26].

All outcome measurements were performed prior to the intervention and after 6 and 12 weeks of intervention.

### 2.4. Statistical Analysis

Data were analyzed using SPSS Statistics for Windows software, version 22.0 (IBM). All data were tested for normal distribution using the Shapiro-Wilk test. Since all of the data were normally distributed, parametric analysis was used for the statistical analysis. The Student’s *t*-test was used for baseline group comparisons. A 2-factor mixed-design ANOVA was used to evaluate the difference between the baseline, mid-test, and post-intervention data in each group, and between groups. The homogeneity between assessments was examined by performing Mauchly’s sphericity test, and the Greenhouse-Geisser correction was used to calculate significant main effects when Mauchly’s sphericity test revealed non-homogeneity of variance (*p* < 0.05). Where significant main effects were observed, a post-hoc Fisher’s least significant difference (LSD) test was used for multiple comparison purposes. A *p* < 0.05 was considered significant.

## 3. Results

The demographic characteristics of the 32 participants are shown in Table 1. The mean age was 69.81 ± 6.65 and 69.75 ± 7.84 years for the OEP and CT groups, respectively. The OEP group consisted of 15 females and 1 male, while the CT group consisted of 14 females and 2 males. There was no statistical difference in age, gender, height, weight, and BMI between the two groups. All participants completed the program with an adherence rate of 100%.

In the present study, SLS and mCTSIB were employed for static balance measurement. As shown in Table 3, for SLS, there was no significant difference between the OEP and CT groups at baseline. We observed a gradual, significant decrease in the stability index, which indicated balance was improving in the OEP group *p* < 0.05 (pre-test = 1.64, mid-test = 0.93, and post-test = 0.62), while the CT group showed a significant decrease in the stability index at post-test (pre-test = 1.63, post-test = 1.23). Comparison between the groups found that the OEP group had a significantly lower stability index compared with the CT group at both mid-test (0.93 vs. 1.38) and post-test (0.62 vs. 1.23), respectively. Because balance is a complex process involving both physiological and cognitive function, we employed the mCTSIB test in our study. A significant decrease in the sway index was present in the OEP group after intervention, but not in the CT group. Comparison between the groups revealed that no difference was observed at baseline; however after intervention the OEP group had significantly lower body sway scores compared with the CT group.

Dynamic balance determined by the BBS showed that the OEP group significantly improved their BBS score after completion of the intervention. A significant increase was observed when comparing post-test vs. pre-test (52.13 vs. 49.25, *p* < 0.05) and post-test vs. mid-test (52.13 vs. 50.19, *p* < 0.05), respectively. Such observation was not observed in the CT group. There was no difference in the BBS scores of the OEP and CT groups.

Proactive balance determined by the TUG test showed that the OEP group had a significant increase in TUG performance at mid-test *p* < 0.05. The post-test data showed a significant difference compared with pre- and mid-test scores (pre-test = 10.83, mid-test = 9.48, and post-test = 8.99). A similar difference in TUG performance was not observed in the CT group. We also found a significant difference in TUG scores between the OEP and CT groups at mid- (9.48 vs. 11.30) and post-test (8.99 vs. 10.91), respectively.

Reactive balance determined by the LOS test showed that the OEP group had a gradual, significant increase in their LOS score *p* < 0.05 (pre-test = 26.31, mid-test = 41.81, and post-test = 59.31), while the CT group had a significantly different score when comparing post-test and mid-test (37.04 vs. 30.19). A clear improvement was demonstrated, with the OEP group having a significantly higher LOS score at both pre- and post-test.

Perceived balance determined by the FES-I found that the modified-OEP was potent in reducing the fear of falls. We observed a gradual, significant decrease in the fear of falls score of the OEP group (pre-test = 24.25, mid-test = 21.75, and post-test = 19.0). There was no significant change in the fear of fall score in the CT group. Comparison between groups demonstrated a significant difference between the OEP and CT groups at both mid- (21.75 vs. 24.13) and post-test (19.0 vs. 24.79).

Balance impairment has been reported as the main cause of falls [6]. The benefits of the OEP on balance performance leading to alleviating falls risk have been well documented [10]. Walking is among the most prevalent physical activities in older adults that is effective in improving lower extremity muscle strength and balance [18]. According to the guidebook and recent literature [11], it is suggested that an additional walk performed as a group results in participants receiving a greater balance advantage. Our present study introduced a modified-OEP by incorporating walking, and conducted the program as a group, to investigate the effects of such a program on balance components.

The modified-OEP could improve static balance, which was consistent with previous reports [20,21], as was shown by the significant decrease in the stability index of participants, measured by the SLS. In addition, the body sway index measurements, according to the mCTSIB, decreased significantly after 12 weeks of intervention. The stability index and body sway index scores of the intervention group were statistically different when compared with the CT group. Accordingly, the modified-OEP is effective for static balance improvement.

The modified-OEP could significantly improve dynamic balance as was seen by the increase in BBS scores from 49.25 (pre-test) to 52.13 (mid-test). The results are in line with previous studies showing that the OEP can improve BBS scores by approximately three [20,21]. The fact that there was not a statistical significance between the OEP and CT groups may be attributed to this study recruiting participants with high BBS scores at baseline, compared to previous literature. According to Rejesky et al., baseline values naturally affect improvements in functional performance, with participants with the weakest baseline performance achieving greater gains [27]. The additional walk incorporated into one OEP session is expected to improve dynamic balance. However, this study did not use the original OEP group outline, thus, the advantage of an additional walk on dynamic balance requires further exploration. Collectively, the modified-OEP could improve dynamic balance.

## 4. Discussion

The modified-OEP could significantly improve proactive balance, as was seen by the increase in TUG performance. The TUG score of the OEP group decreased by approximately 2 s when comparing pre- and post-test data. To date, it remains unclear whether the OEP performed in a shorter period, <12 weeks, can improve TUG performance. While one study reported crucial benefit [28], others found no change [9,29]. Our results demonstrated the TUG improvement was significantly observed in as little as six weeks (mid-test). According to a previous report [30], an individual who has a TUG score of 13.5 or higher is at risk of falls. The participants recruited in this study had low TUG scores, indicating good balance performance. Previous literature reported that walking exercise could improve lower limb function and TUG scores [18]. Presumably, the walking exercise incorporated into one OEP session might enhance lower limb function, e.g., increase muscle mass, resulting in significantly improved TUG performance. However, the precise mechanism requires further exploration. Collectively, the modified-OEP could improve proactive balance.

The modified-OEP could significantly improve reactive balance as was seen by the overall increase in LOS score, indicating better directional control. The improvement in LOS score was observed in as little as 6 weeks and continued increasing at week 12 in the OEP group. We also found a significant improvement in LOS score in the CT group at week 12. However, since there was no trend of increase, we assume that such an increase may be due to the learning effect. To our knowledge, LOS has not been employed to determine the effect of the OEP. The LOS is suitable for reactive balance assessment as reported previously [22], therefore it was employed in our study. It remains unclear whether LOS score is significantly linked to the risk of falls [31]. However, a high LOS score indicates good stability, which relates to falls [32], thus, this enhancement should mitigate the risk of falls. Accordingly, the modified-OEP is effective for reactive balance improvement.

Previous literature reported a strong correlation between actual and perceived balance [14]. We employed the FES-I test to measure fear of falling. Fear of falling causes psychological issues that limit activity and impair physical functions. Impaired physical function, in turn, affects the neuromuscular ability and increases the risk of falling and the fear that comes with it, perpetuating a vicious cycle [33]. Thus, an intervention that benefits fear of falling is considered a key for falls prevention. Consistent with the original OEP [26,28], a significant decrease in FES-I score was observed in the OEP group but not in the CT group, suggesting the modified-OEP enhances movement confidence, which would result in maintaining physical activity.

A couple of unexpected outcomes were presented in our study. We found significant improvement in the SLS and LOS scores in the control group (Table 3) that received no intervention. These observations may be attributed to measurement reactivity, which refers to a condition in which the act of measuring causes changes in the people being measured [34]. As anticipated, this process resulted in a small increase in the physical activity of participants in the control group [34]. Waters et al., suggested that changes in the physical activity of a control group are most prevalent when the assessment is conducted over a long period of time [35]. This explanation could apply to our study, since SLS and LOS scores significantly improved at 12 weeks but not 6 weeks following the intervention.

The strength of this study is that we not only demonstrated the advantages of the modified-OEP on balance, but also provided further insight into balance components. A number of studies reveal poor correlation among the four components of actual balance [36,37]. Due to the task-specific nature of balance, specific balance categories should be evaluated separately [13]. Accordingly, the modified-OEP introduced in this study could improve all four components of actual balance, and perceived balance. Our report could additionally underline the association between actual and perceived balance, as both increased. In addition, the assessment of outcomes conducted after a 6- and 12-week intervention period revealed both the short- and long-term effects of the modified-OEP.

This study has some considerable limitations. First, the majority of participants in each group were female, which probably makes the results not applicable to all genders. According to Ansdell et al., gender differences through physiological system phenotypes lead to distinct responses to exercise. For instance, females have a higher proportion of type 1 muscle fibers resulting in more fat oxidization but less in protein and carbohydrate than those of males [38]. The respiratory efficacy is different because females have smaller lungs than males, even if their height is matched [39]. We were unable to perform a separate analysis by gender in this study due to the small number of male participants in each group (one in the modified-OEP group and two in the CT group). Second, we did not include the original OEP in our study. Therefore, it remains inconclusive whether our modified-OEP is at least as effective as the original program. According to a systematic review by Martins et al. [40], various formats of modified-OEPs have been proposed to date, including multisensory balance exercise, group exercise, and DVD delivery. All demonstrated positive balance and fall prevention benefits. It is unclear, however, if the modified format is more effective than the original, and which modified format is better [40]. Another limitation is that the range of participant ages was wide, since we recruited participants aged between 60–85 years old. According to previous literature, muscle strength, balance, and mobility decline significantly after the age of 70 [41,42]. Consequently, this could result in a significantly different improvement after the intervention, with participants with poor baseline performance achieving greater gains in their functional performance [27]. Given the lack of statistical significance between age and baseline values of all outcome measurements, the wide age range does not appear to have affected the results of this study. Additionally, the number of participants recruited in this study was relatively low, resulting in small effect sizes of most outcome measurements.

In conclusion, this study reported the benefits of a modified-OEP that included walking exercise in one session, on the four components of actual balance, and perceived balance. Further investigation should address whether this modified-OEP is beneficial in large populations with more balanced gender proportions, as well as compare the effects of this program to those of the original OEP.

## Figures and Tables

**Table 1:** Demographic characterization of modified-Otago Exercise Program (OEP) and control groups at baseline.

Variables	OEP (*n* = 16)		CT (*n* = 16)		*p*-Value
	Mean	Std. Deviation	Mean	Std. Deviation	
Age, years	69.81	6.65	69.75	7.84	0.434
Gender					
Female, % (*n*)	93.75(15)		87.5(14)		0.544
Male, % (*n*)	6.25(1)		12.5(2)		
Height (cm)	155.44	6.77	156.81	6.12	0.729
Weight (kg)	56.31	11.98	57.25	9.60	0.855
BMI	23.24	3.50	23.28	3.44	0.842

**Table 2:** Features of the modified-OEP.

Program			Activity	Intensity	Progressions	Duration	Frequency
Modified-OEP (this study)		Walking	Walking with correct pattern by physical therapist suggestion in the training room	Self-selected pace	-	Fifteen minutes	Three times a week (before original OEP 30 min)
	Original OEP [8]	Strengthening	Five leg muscle strengthening exercises; four levels of difficulty	Moderate; 8–10 repetitions before fatigue	Increase from one to two sets. Increased amount of ankle weight after 2 sets of 10	Approximately 30 min total for exercises; exercises can be divided up over the day	At least three times a week
		Balance	Twelve balance retraining exercises; four levels of difficulty	Moderate; Each exercise at a level that the individual can safely perform unsupervised	Supported exercise to unsupported exercise		
		(Optional) Walking	Advice about walking	Usual pace with usual walking aid	Walk indoors. Advance to walk outdoors when strength and balance improved	Thirty minutes; can be split into three ten-minute walks throughout the day	At least twice a week
		Walking	Walking with correct pattern by physical therapist suggestion in the training room.	Self-selected pace	-	Fifteen minutes	Three times a week (after original OEP 30 min)

**Table 3:** Effect of the Modified-OEP on balance components.

Balance Components	Assessment		OEP (*n* = 16)						CT (*n* = 16)									Effect Size
			Pre-Test		Mid-Test		Post-Test		Pre-Test		Mid-Test		Post-Test		ANOVA Analysis *p* Value			
			Mean	Std. Deviation	Mean	Std. Deviation	Mean	Std. Deviation	Mean	Std. Deviation	Mean	Std. Deviation	Mean	Std. Deviation	Time	Group	Time × Group	
Static	SLS		1.64	0.89	0.93 ^a^*	0.33	0.62 ^bc^*	0.17	1.63	0.68	1.38	0.54	1.23 ^b^	0.55	0.000	0.028	0.029	0.111
	mCTSIB	EO-firm	0.63	0.27	0.47 ^a^*	0.09	0.47 ^b^*	0.12	0.58	0.26	0.64	0.27	0.69	0.36	0.579	0.111	0.009	0.146
		EC-firm	1.17	0.36	1.03	0.32	0.88 ^b^*	0.26	1.01	0.36	1.1	0.36	1.19	0.55	0.783	0.418	0.021	0.121
		EO-foam	1.09	0.4	0.86	0.16	0.75 ^bc^*	0.2	1.04	0.42	1.03	0.38	1.15	0.64	0.095	0.155	0.003	0.182
		EC-foam	2.74	0.58	2.51	0.54	2.05 ^bc^*	0.5	2.85	1	2.95	1.13	2.91	1.18	0.087	0.079	0.044	0.102
Dynamic	BBS		49.25	2.86	50.19	2.88	52.13 ^bc^	2.66	50.38	3.32	50.44	3.61	50.23	3.28	0.008	0.864	0.003	0.196
Proactive	TUG		10.83	1.54	9.48 ^a^*	1.36	8.99 ^bc^*	1.09	11.19	1.57	11.30	1.65	10.91	1.17	0.000	0.005	0.000	0.282
Reactive	LOS		26.31	7.48	41.81 ^a^*	8.8	59.31 ^bc^*	12.38	33.75	13.42	30.19	15.71	37.04 ^c^	12.63	0.000	0.022	0.000	0.510
Perceived	FES-I		24.25	5.47	21.75 ^a^	4.59	19.0 ^bc^*	3.39	22.87	6.84	24.13	5.41	24.79	6.43	0.110	0.201	0.001	0.275

SLS = Single-leg Stand test, mCTSIB = modified Clinical Test of Sensory Interaction and Balance, EO—firm = Eyes open—firm surface condition, EC—firm = Eyes closed—firm surface condition, EO—foam = Eyes open—foam surface condition, EC—foam = Eyes closed—foam surface condition, BBS = Berg Balance Scale, LOS = Limits of Stability test, and FES-I = the Falls Efficacy Scale International (FES-I). A 2-factor mixed-design ANOVA was used for statistical analysis. A *p* < 0.05 was considered significant. ^a^ = significant between pre-test and mid-test. ^b^ = significant between pre-test and post-test. ^c^ = significant between mid-test and post-test. * = significant between groups at the same time.

## Data Availability

Not applicable.
